# Veterinary Department, University of Pennsylvania

**Published:** 1890-06

**Authors:** 


### VETERINARY COLLEGE NOTES.

*Veterinary Department, University of Pennsylvania.*—The following twenty-two gentlemen have passed their final examinations and will receive their diplomas conferring the degree of *Veterinariæ Medicinæ Doctor* (V.M.D.), on June 5th: Harry Bannister, Philadelphia; Nathan A. Cohen, Philadelphia; Charles A. Dohan, Philadelphia; Harry L. Eddy, Minneapolis, Minn.; John M. Eshleman, Fagg's Manor, Pa.; John W. Harrigan, Philadelphia; Charles Rudolph Jolly,* Philadelphia; Thomas J. Kean, Lock Haven, Pa.; Edgar H. Landes, Philadelphia; Samuel D. Larzelere, Willow Grove, Pa.; John J. Maher, Philadelphia; Harry A. Meisner, Baltimore, Md.; Mayhew Michener, Colmar, Pa.; Harry C. Millar, Hatboro, Pa.; Edwin S. Muir, Philadelphia; Charles F. Oat, West Chester, Pa.; Leonard Pearson, Ithaca, N. Y.; Thomas Raynor, Philadelphia; Frank L. Smith, Philadelphia; Edgar Tully, Philadelphia; John P. Turner, West Chester, Pa.; Jeremiah P. Zuill, Bermuda, W. I.

* Being under the required age, will not receive his diploma until a later commencement.

